# Anti-spermatogenic activities of *Taraxacum officinale* whole plant and leaves aqueous extracts

**Published:** 2016-06-15

**Authors:** Lubna Hamid Tahtamouni, Rema Ahmad Al-Khateeb, Reem Nasser Abdellatif, Zainab Ali Al-Mazaydeh, Salem Refaat Yasin, Samer Al-Gharabli, Ali Zuhair Elkarmi

**Affiliations:** 1 *Department of Biology and Biotechnology, Faculty of Science, The Hashemite University, Zarqa, Jordan; *; 2 *Department of Pharmaceutical and Chemical Engineering, School of Applied Medical Sciences, German Jordanian University, Amman, Jordan.*

**Keywords:** Dandelion, Maturation arrest, Rat, Renewal, Spermatogenesis

## Abstract

*Taraxacum officinale* has been used in Jordan folk medicine to treat male infertility. A recent study has proved a contradictory effect of the whole plant aqueous extract. The aim of the current study was to determine if the leaves of *T. officinale *have similar anti-fertility activities, and whether this effect is mediated through the regulation of spermatogonial stem cells (SSCs). Fifty adult male rats were divided into five groups. Two groups were gavaged with 1/10 of LD_50_ of* T. officinale* whole plant (1.06 g kg^-1^ body weight) or leaves (2.30 g kg^-1^ body weight) aqueous extract; while two groups were gavaged with 1/20 of LD_50_ of *T. officinale* whole plant (2.13 g kg^-1^) or leaves (4.60 g kg^-1^) extract. The control group received distilled water. Oral administration of *T. officinale* (whole plant and leaves aqueous extract) caused a significant decrease in testis and seminal vesicle weight, a reduction in serum testosterone concentration, impaired sperm parameters, and a decrease in pregnancy parameters. Testicular histology of treated rats showed structural changes such as hypoplasia of germ cells, reduction in the thickness of germinal epithelium, arrest of spermatogenesis at spermatid stage (late maturation arrest) and reduction in the number of Leydig cells. Gene expression levels of two SSCs markers (GFRα1 and CSF1) responsible for self-renewal were relatively counter-balanced. In conclusion, *T. officinale* whole plant and leaves aqueous extracts changed the gene expression of two SSCs markers leading to the imbalance between spermatogonia self-renewal and differentiation causing late maturation arrest.

## Introduction

Spermatogenesis is the process of sperm production; it begins at puberty and is maintained throughout the life of mammals due to the presence of spermatogonial stem cells (SSCs).^1^ The SSCs are located on the basement membrane of the seminiferous tubules;^2^ they originate from primordial germ cells (PGCs) which arise from the endoderm of the yolk sac wall. The PGCs migrate to the genital ridge; and differentiate into gonocyte which become enclosed by Sertoli cells to form seminiferous tubules. Gonocytes proliferate and arrest in Gap0/Gap1 phase until birth, after which all of the gonocytes will be converted to SSCs.^3^

The SSCs either divide into two new single SSCs or into a single spermatogonia (As) which are capable of either self-renewal or differentiation into two A paired (Apr) spermatogonia. Apr spermatogonia do not complete cytokinesis and remain joined by intercellular bridges; the mitotic division of Apr spermatogonia gives rise to A aligned (Aal) spermatogonia that form chains of 4, 8 or 16 aligned spermatogonia. The Aal spermatogonia differentiate into A1, A2, A3, A4, intermediate and B spermatogonia. B spermatogonia will in turn undergo mitosis and meiosis to produce mature sperm.^4^

The different stages of SSCs are controlled by specific markers which are also used to distinguish between these stages. Three of the most important markers required to regulate the proliferation and differentiation of SSCs are Glial Cell-derived neurotrophic factor (GDNF), colony-stimulating factor 1 (CSF1) and promyelocytic leukemia zinc-finger (PLZF). The GDNF is a member of the transforming growth factor β (TGF-β) superfamily produced by Sertoli cells under the control of follicle stimulating hormone (FSH)^.^^5^ The GDNF is responsible for the maintenance and self-renewal of SSCs,^6^ its action is mediated through its receptors GDNF family receptor alpha 1 (GFR1α) and rearranged during transformation (RET; tyrosine kinase transmembrane protein).^7^

The CSF1 is secreted by Leydig cells and myoid cells.^8^ CSF1 receptor is restricted to the undifferentiated spermatogonia (As, Apr, and Aal) expressing the cell surface antigen THY1, and it plays an important role in the proliferation of spermatogonial stem cells.^9^ The PLZF is one of the first transcription factors required for self-renewal of SSCs and is restricted to As, Apr and Aal.^10^

People of different cultures and places have used folk medicine for the treatment of certain medical problems such as liver illness, diabetes, inflammation and infertility.^11^ Several plants such as *Taraxacum officinale* (L.) Weber ex F.H. Wigg. (Compositae) have been traditionally used in Jordan folk medicine to treat infertility. However, a recent study has proved conflicting effect of the whole plant aqueous extract which acted as an anti-spermatogenic agent rather than an enhancer of fertility.^12^

Therefore, the aim of this study was to determine if the leaves of *T. officinale *have the similar anti-fertility activities, and whether these anti-spermatogenic effects are mediated through spermatogonial stem cell regulation. 

## Materials and Methods

***Taraxacum officinale*** **collection and extraction.** *Taraxacum officinale* whole plant and leaves were collected during the flowering season (February to October) of 2014 from Amman, Sukhna region and the campus of the University of Jordan, (Amman, Jordan). The plant was authenticated by a plant taxonomist, and a voucher specimen was deposited at the herbarium (HU-42741), Department of Biology and Biotechnology, The Hashemite University. *Taraxacum officinale* fresh leaves and whole plant were washed under running water; air dried (away from the sun) and then ground. The aqueous extract of the whole plant or leaves was prepared by adding 1 L of distilled water to 100 g of dried plant materials at 45 ˚C for two days. The mixture was then filtered to remove any particulate matter followed by lyophilization. The resulting powder, 21.40 g of leaves (21.40% w/w) and 22.20 g of whole plant (22.20% w/w), was stored until use.^12^^-^^14^

**Screening of the chemical constituents of the aqueous extract of** ***Taraxacum officinale*** **whole plant or leaves** **. **High Performance liquid chromatography-mass spectroscopy (HPLC-MS; Finnigan surveyor PDA plus detector coupled with ESI single quad mass spectrometer; Thermo Finnigan, San Jose, USA) was used to determine the chemical composition of* T. officinale*. 

**Animal Treatment** **.** Fifty adult Wistar male rats (*Rattus norvegicus*) weighing between 150 to 200 g were randomly selected from the animal house, The Hashemite University (Zarqa, Jordan). Animals were housed individually in cages and maintained under standard conditions (12 hr light/12 hr dark cycle; 25 ˚C); standard laboratory food and tap water were given *ad libitum*. After one week of acclimatization, the rats were randomly divided into five different groups. The animals of the first and second groups were gavaged with 1/20 and 1/10 of LD_50_ of* T. officinale* whole plant aqueous extract (low dose-receiving group, LDWP, and high dose-receiving group, HDWP, respectively).^12^ The animals of the third group were gavaged with 1/20 of LD_50_ (2.30 g kg^-1^ body weight) of* T. officinale* leaves aqueous extract and were considered as the low dose-receiving group (LDL)*. *The fourth group received 1/10 of LD_50_ (4.60 g kg^-1^ body weight) of* T. officinale* leaves aqueous extract and was considered as the high dose-receiving group (HDL). The fifth group of animals was gavaged with distilled water and was considered as control group. The doses were given orally using a gavage needle for 60 consecutive days.^12^^,^^13^

**Fertility test.** On day 55, each male rat from each group was mated individually with two fertile proestrus females for five days. Successful mating was confirmed by the presence of sperm in the vaginal smear. The females were then separated until the end of the pregnancy period. Number, weight and sex of offspring were recorded.^13^^,^^15^

**Collection of samples.** Male rats were anesthetized and weighed; afterwards the rats were sacrificed by cervical dislocation. Testes, seminal vesicles, kidneys, and liver were collected and weighed. Blood was collected in plain blood tubes by heart puncture and serum was separated by gravity and stored at – 20 ˚C until use. Serum was used to analyze testosterone level (Immulite 1000 immunoassay system; Siemens, Berlin, Germany).

**Sperm analysis.** The cauda epididymis of each rat was sliced open in 3 mL fresh Hank’s balanced salt solution (HBSS; Sigma-Aldrich, St. Luis, USA), and was incubated for 5 min at 37 ˚C to allow sperm to swim out of the epididymal tubules. After which, the epididymal fluid was centrifuged at 500 rpm for 3 min and the supernatant was analyzed. Cauda epididymal suspension was diluted 1:10 with fresh HBSS, and 10 μL were applied to the Neubauer’s counting chamber to evaluate sperm count (10^3^ per mL) and motility (percentage).^16^ A volume of 10 μL of sperm suspension were smeared on a clean glass slide and air dried. Sperm smear was fixed in methanol for 20 min, hydrated in alcohol series, stained with hematoxylin for 1 to 2 min and then with eosin for 1 min. Then, the smear was dehydrated in alcohol series, cleared in xylene for 10 min and mounted with a mixture of distyrene, a plasticizer, and xylene (DPX). A total number of 200 sperms were examined per each slide and classified into morpho-logically normal or abnormal.^17^ Sperm chromatin DNA damage was assessed by the acridine orange (AO) staining method as described previously by Tejada *et al.* and through agarose gel electrophoresis.^18^

**Histological studies** **. **The right testis was cut into small pieces (5 × 5 mm) and placed in Bouin’s fixative (Sigma-Aldrich) for 24 hr. The samples were then transferred into 70.00% ethanol (Sigma-Aldrich), while the excess picric acid was removed by adding few drops of saturated lithium carbonate. The samples were dehydrated in a series of ethanol, cleared in xylene (Sigma-Aldrich), followed by embedding in paraffin wax. Sections (5 to 7 µm thick) were mounted on clean glass slides, stained with hematoxylin and eosin (H & E), and mounted in DPX. Finally, the sections were examined by Nikon Eclipse 50i microscope (Nikon, Tokyo, Japan).

**Immunoblotting.** Immunoblotting of testicular proteins against GFRα1, PLZF and CSF1 antibodies was performed according to Sambrook *et al.*^19^ Briefly, half of the left testis was lysed in cold lysis buffer (2.00% sodium dodecyl sulfate, 10 mM Tris pH 7.5, 10 mM NaF, 2 mM EGTA, 10 mM dithiothreitol). Proteins were resolved by electrophoresis on 10.00% SDS-polyacrylamide gels, transferred onto nitrocellulose membranes, blocked using 1.00% (w/v) bovine serum albumin (BSA; Bio Basic, Ontario, Canada) in Tris-buffered saline (TBS; Bio Basic), and exposed overnight at 4 ˚C to the primary antibodies, mouse monoclonal anti-PLZF antibody (1:200; Abcam, Bristol, UK), rabbit polyclonal anti-CSF1 antibody (1:200; Abcam), rabbit polyclonal anti-GFRα1 antibody (1:200; Abcam), and mouse monoclonal anti-actin antibody (1:1000; Sigma-Aldrich). After washing and incubation with appropriate secondary antibodies conjugated to horseradish peroxidase, the membranes were washed and the bands were visualized using the 3,30,5,50 tetra methyl benzidine system (TMB; Sigma-Aldrich). Signals were quantified using TotalLab software (version 2.0; TotalLab, Newcastle, UK).

**Real-time PCR** **.** To determine the quantity of GFRΑ1, PLZF and CSF1 mRNA, real-time PCR (RT-PCR) was used.^20^ After RNA extraction, cDNA was prepared using Power cDNA Synthesis Kit (iNtRON Biotechnology, Seoul, Korea). Amplification of target cDNA for the different stem cell markers and actin (as a house keeping gene) was done using KAPA SYBR FAST qPCR Kit Master Mix (Kapa Biosystems, Wilmington, USA) on LineGene 9680 (Bioer Technology, Zhejiang Province, China). A volume of 5.00 µL of cDNA were mixed with 0.50 µL of forward primer (25×), 0.50 µL reverse primer (25×),^5^^,^^21^ 5.50 µL nuclease free water and 12.50 µL master mix. Quantitative RT-PCR was performed under the following conditions: hold stage at 95 ˚C for 3 min, followed by 40 cycles of PCR stage at 95 ˚C for 3 sec, 60 ˚C for 20 sec and 72 ˚C for 30 sec. Each RT-PCR reaction was run as duplicate for each primer. 

**Ethical aspects.** The study was approved by the Institute Review Board (IRB) of The Hashemite University. Animal care, handling, and all of the experiments performed were approved by The Hashemite University Institutional Animal Care and Use Committee.

**Statistical analysis.** Statistical analysis of data was performed using STATISTICA analysis program (version 7.0; StatSoft Inc., Tulsa, USA). In order to determine differences among three or more means, one way analysis of variance with Fisher's LSD for multiple comparisons post-tests were performed. Data are expressed as mean ± SD and the level of significance was set as *p *< 0.05.

## Results

**Fig. 1:**
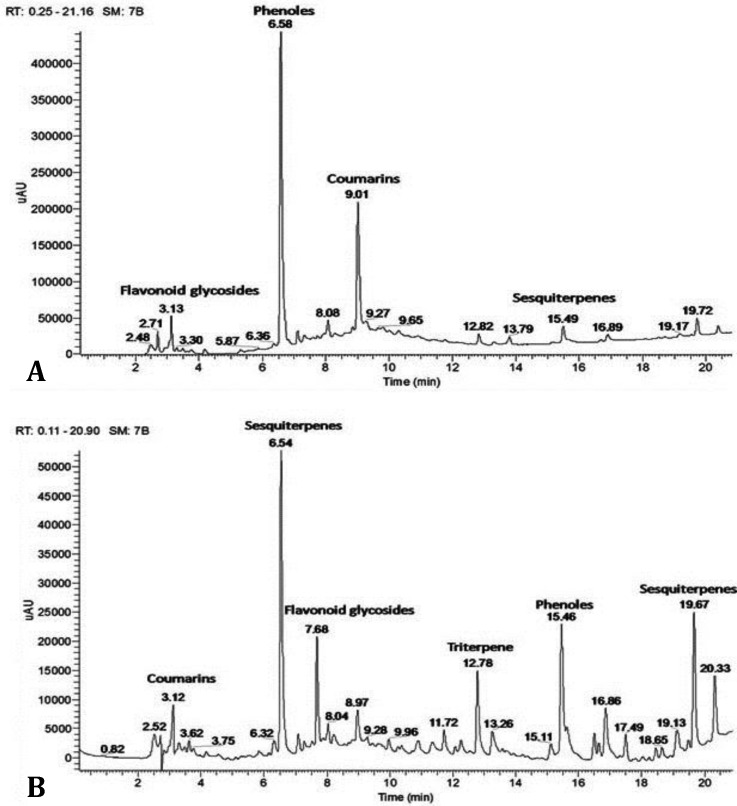
**)** HPLC-MS chromatogram of *T. officinale* leaves aqueous extract; **B)** HPLC-MS chromatogram of *T. officinale* whole plant aqueous extract

**HPLC-MS screening of the chemical constituents of** ***Taraxacum officinale*** **whole plant and leaves aqueous extract** **s.** High performance liquid chromatography-mass spectroscopy (HPLC-MS) analysis of *T. officinale* leaves aqueous extract revealed several peaks corresponding to various compounds such as phenols including chlorogenic acid, chicoric acid, hydroxycinnamic acid (caffeic acid), flavonoid glycosides (luteolin 7-O-glucoside, quercetin 7-O-glucoside), sesquiterpenes (taraxinic acid -D-gluco- pyranoside, 11,13-dihydrotaraxinic-acid), and coumarins (cichoriin, aesculin), (Fig. 1A). The HPLC-MS analysis of *T. officinale* whole plant revealed the presence of several constituents such as phenols (chlorogenic acid, chicoric acid, caffeic acid, monocaffeoyltartaric acids), flavonoid glycosides (luteolin 7-O-glucoside, quercetin 7-O-glucoside, luteolin 7-O-rutinoside, luteolin 7-diglucosides), sesquiterpenes (tetrahydroridentin B, taraxacolide-O--glucopyranoside, ixerin D, ainslioside, 11-13-dihydro-lactucin, taraxinic acid -D-glucopyranoside, 11,13-dihydrotaraxinic-acid), triterpene (taraxasterol, and amyrin) and coumarins (scopoletin, esculetin, cichoriin, aesculin), (Fig. 1B). Mass spectroscopy was used to identify the peaks presented in the HPLC chromatograms (Fig. 1). The masses of the compounds found in the extracts were matched with known compounds which have been identified previously in *T. officinale*.^22^ For simplicity, peaks were assigned as a group of chemicals (Fig. 1) where the isolation of pure compounds was not the scope of the current work.

***Taraxacum officinale*** **whole plant or leaves aqueous extract treatment reduces testis and seminal vesicle weights. **The initial and final weights of rats were recorded before and after the 60 days of treatment. The increase in body weight gain of treated animals was insignificant when compared with the control group (Table 1). The weight of the testes, seminal vesicles, kidneys and liver of control and treated groups were recorded on the day of sacrifice (Table 1). The results showed that the testes weight of rats treated with HDWP or leaves decreased significantly when compared with the control and LDWP-receiving group (Table 1). The weight of the seminal vesicles showed a significant decrease in all of the treated groups in comparison with the control group (Table 1). The kidney and liver weights of the treated groups showed an insignificant decrease in comparison to the control group (Table 1).

***Taraxacum officinale*** **whole plant or leaves aqueous extract decreases serum testosterone concentration.** *Taraxacum officinale* administration caused a decrease in testosterone concentration in all of the treated groups when compared with the control group and in the HDL-treated group when compared to the LDWP-treated group (Table 2).

**Fig. 2:**
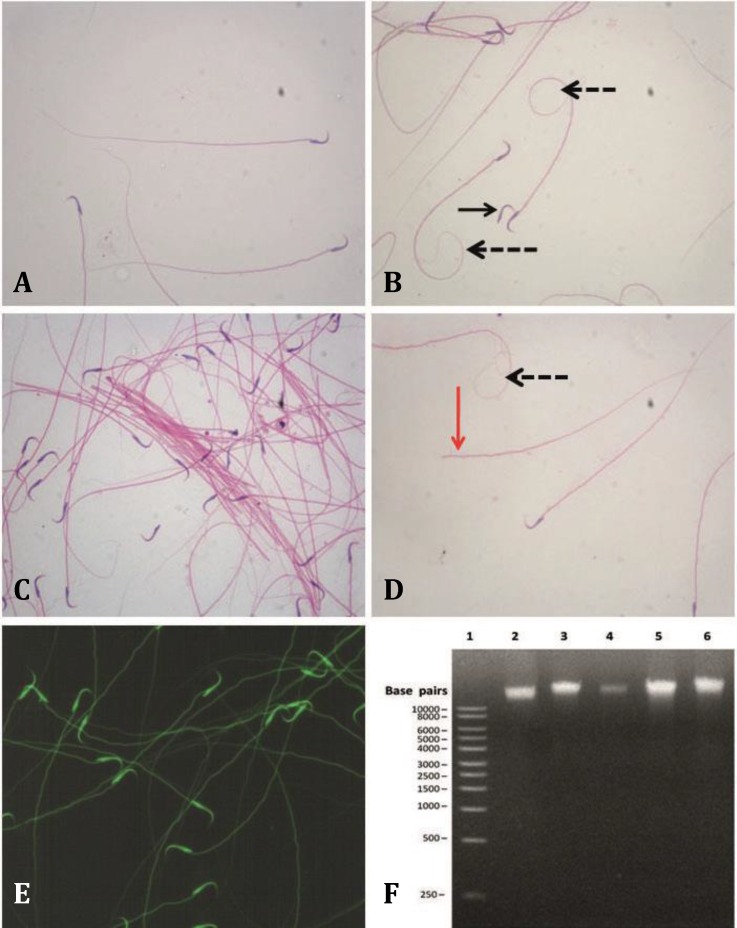
Effect of *T. officinale* treatment on rat sperm morphology and chromatin integrity. **A)** Normal rat sperm showing the hooked head and tail. **B)** Coiled-tail sperm (dashed arrow) and tailless sperm (arrow). **C)** Sperm agglutination. **D)** Tailless sperm (red arrow) and coiled-tail sperm (dashed arrow). **E)** Evaluation of chromatin integrity by acridine orange staining. All sperms showed green fluorescence indicating integrated chromatin. **F)** Representative agarose gel electrophoresis analysis of sperm DNA of control and treated rats. Lane 1: 1 kb DNA molecular weight marker; Lane 2: Control; Lane 3: LDWP; Lane 4: HDWP;  Lane 5: LDL; Lane 6: HDL

**The Effect of** ***T. officinale*** **whole plant or leaves aqueous extract on cauda epididymal sperm parameters** **.** Cauda epididymal sperm count (10^3^ per mL) in all treated groups showed a significant decrease when compared with the control group (Table 2). The results also showed a significant decrease when comparing between the LDWP-receiving group and the leaves-receiving groups (Table 2). On the other hand, the percentage of progressively motile sperms in all treated groups showed a significant decrease in comparison to the control group and in the leaves-receiving groups in comparison to the LDWP-treated group (Table 2). Different sperm morphological abnormalities such as coiled tail, headless sperm, tailless sperm and aggregations were observed in control and treated groups (Figs. 2A to 2D). The percentage of sperms with morphological abnormalities in the treated groups increased significantly in comparison with the control group (Table 2), the increase was also significant when comparing the whole plant-receiving groups with the leaves- receiving groups (Table 2).

**Table 1:** Body weight gain and organ weight (g) in control and treated groups. Values are expressed as mean ± SD, n = 10 in each group.

**Groups**	**Weight gain**	**Testis**	**Seminal vesicle**	**Liver**	**Kidney**
**Control **	80.20 ± 13.70	1.80 ± 0.20	1.20 ± 0.30	10.70 ± 0.40	1.10 ± 0.20
**LDWP **	95.30 ± 17.20	1.70 ± 0.20	1.00 ± 0.10^a2^	9.90 ± 1.00	1.00 ± 0.20
**HDWP**	82.60 ± 21.10	1.40 ± 0.30^ab^	0.90 ± 0.10^a2^	10.20 ± 1.30	0.90 ± 0.20
**LDL **	93.00 ± 33.60	1.50 ± 0.10^ab^	0.80 ± 0.10^a2^	9.70 ± 1.50	0.90 ± 0.10
**HDL**	84.90 ± 33.30	1.40 ± 0.10^ab^	0.70 ± 0.20^a3^	9.50 ± 0.90	0.90 ± 0.10

LDWP: Low dose-receiving whole plant; HDWP: High dose-receiving whole plant; LDL: Low dose-receiving leaves; HDL: High dose-receiving leaves. a: *p* < 0.05; a2: *p* < 0.01; a3: *p* < 0.001 compared to control, b: *p* < 0.05 compared to LDWP.

**Table 2:** Effect of *Taraxacum officinale* whole plant or leaves aqueous extract on testosterone concentration and sperm parameters. Values are expressed as mean ± SD, n=10 in each group

**Groups**	**Testosterone concentration (ng mL** ^-1^ **)**	**Sperm count (10** ^3^ ** mL** ^-1^ **)**	**Sperm motility (%)**	**Sperm abnormality (%)**
**Control**	4.20 ± 0.40	85.20 ± 9.20	67.60 ± 8.70	3.90 ± 1.10
**LDWP**	1.90 ± 0.20a3	45.30 ± 7.10a3^d^e2	32.40 ± 5.10a3^d^^e^	21.20 ± 4.90a3c3d3e3
**HDWP**	1.80 ± 0.30a3	42.90 ± 5.70a3^e^	29.10 ± 9.70a2	35.70 ± 7.20a3b3^d^e3
**LDL**	1.80 ± 0.40a3	37.10 ± 5.20a3^b^	24.80 ± 3.90a3b2	41.90 ± 13.60a3b3^c^
**HDL**	1.50 ± 0.20a3^b^	34.10 ± 5.00a3^b^^c^	25.50 ± 3.40a3^b^	46.90 ± 6.80a3b3c2

LDWP: Low dose-receiving whole plant; HDWP: High dose-receiving whole plant; LDL: Low dose-receiving leaves; HDL: High dose-receiving leaves.

a2 : *p* 0.01;

a3 : *p* 0.001 compared to control,

b : *p* 0.05;

b2 : *p* 0.01;

b3 : *p* 0.001 compared to LDWP,

c : *p* 0.05;

c2 : *p* 0.01;

c3 : *p* 0.001 compared to HDWP,

d : *p* 0.05;

d3 : *p* 0.001 compared to LDL,

e : *p* 0.05 ;

e2 : *p* < 0.01,

e3 : *p* < 0.001 compared to HDL.

**Sperm chromatin integrity** **. **Cauda epididymal sperms were smeared and stained with AO. The results indicated that *T. officinale* aqueous extract of both the whole plant and leaves did not cause DNA fragmentation since all of the sperms fluoresced green (Fig. 2E). In addition, DNA extracted from epididymal sperms was analyzed on agarose gel electrophoresis and the results indicated intact DNA (one band) without fragmentation (Fig. 2F).

**Effects of** ***T. officinale*** **whole plant or leaves aqueous extract on pregnancy rate** **.** The results of the fertility test showed that the pregnancy rate of all treated groups, including HDWP (55.00%), LDL (50.00%), and HDL (40.00%), except for the LDWP-receiving group (80.00%), decreased significantly (*p* < 0.05 for HDWP and LDL-receiving groups and *p *< 0.01 for the HDL-receiving group) when compared to the control group (90.00%). In addition, the average number of fetuses delivered for all of the treated groups, including LDWP (7.70%), HDWP (7.20%), LDL (6.50%), and HDL (5.70%), decreased significantly (*p* < 0.05 for the whole plant-receiving groups and *p* < 0.01 for the leaves-receiving groups) in comparison with the control group (8.80%). 

**Fig. 3:**
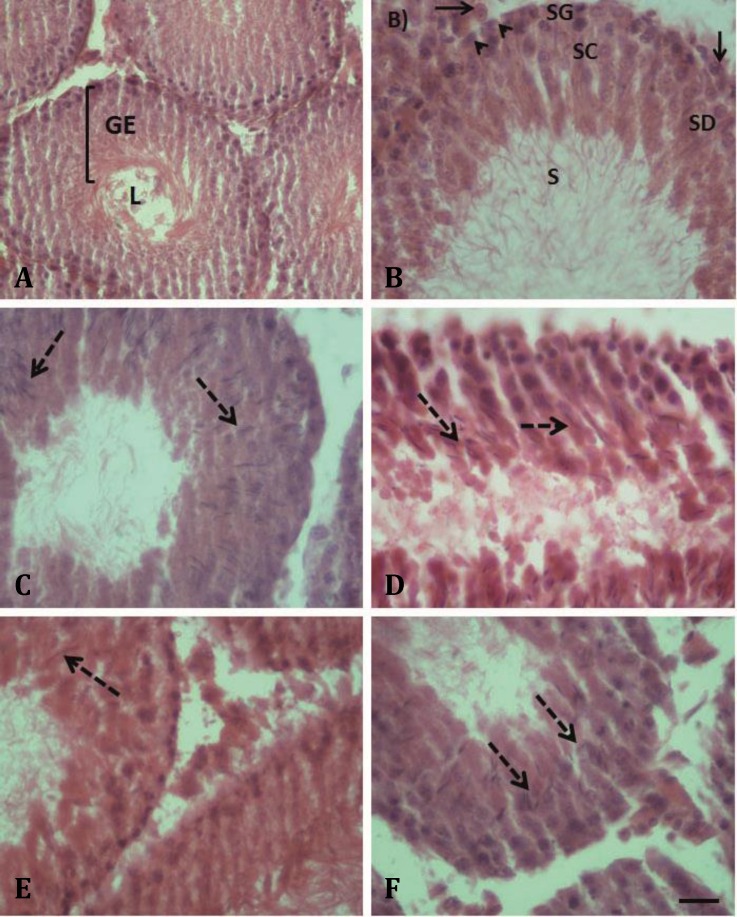
Treatment of male rats with *Taraxucum officinale* whole plant or leaves aqueous extract causes disruption in spermato-genesis.** A, B)** Testicular cross sections from control rat showing seminiferous tubules consisting of lumen (L) and germinal epithelium (GE) and interstitial tissue including Leydig cells (arrow) and myoid cells (arrow head). The seminiferous tubule shows organized germinal epithelium with typical spermatogenic cells: spermatogonia (SG); spermatocytes (SC); spermatids (SD); and sperms (S). **C)** Cross section of seminiferous tubules of LDWP-receiving group showing increased spaces between seminiferous tubules, interstitial tissue hypoplasia, and spermatogenesis late maturation arrest (dashed arrow). **D)** Cross section of semini-ferous tubules of HDWP-receiving group showing germinal epithelium disorganization and late maturation arrest (dashed arrow). **E)** Cross section of seminiferous tubule of LDL-receiving group showing late maturation arrest (dashed arrow) and a reduction in germinal epithelium thickness. **F)** Cross section of seminiferous tubule of HDL-receiving group showing late maturation arrest (dashed arrow) and hypoplasia of germ cells and interstitial tissue (H & E, Scale bar = 50 μm for all panels

**Testicular sections of rats treated with the whole plant or leaves aqueous extract of** ***T. officinale*** **show late maturation arrest.** Testicular cross sections of control rats showed normal histological appearance of semini-ferous tubules; all of the various stages of spermatogenesis starting from spermatogonia to sperms were observed, and the interstitial tissue was intact (Figs. 3A and 3B). Seminiferous tubules of the LDWP-receiving group showed normal and abnormal histology. The abnormal histology appeared as arrest of spermatogenesis at spermatid stage (late maturation arrest; dashed arrow) with fewer sperms in the lumen (Fig. 3C). Testicular sections prepared from HDWP-receiving rats showed disorganization of the germinal epithelium (GE) cells, late maturation arrest and the interstitial tissue (the inter-tubular connective tissue) showed signs of hypoplasia and loss of cells (Fig. 2D). Testicular histology of rats treated with* T. officinale* leaves aqueous extract showed hypoplasia in GE and interstitial tissue, late maturation arrest and large spaces (inter-tubular) between seminiferous tubules (Figs. 3E and 3F). 

In addition, the diameter of the seminiferous tubules and the thickness of the GE of control and treated groups were measured (Table 3). The results indicated a reduction in seminiferous tubules diameter and GE thickness in treated groups when compared to the control and the LDWP- receiving groups (Table 3). The hypoplasia of the interstitial tissue and loss of cells was confirmed by measuring the intertubular distances and the number of Leydig cells per unit area (200 µm^2^), (Table 3). It was found that *T. officinale* treatment resulted in increasing the distances between the seminiferous tubules (intertubular distance) and decreasing the number of Leydig cells per 200 µm^2^ when compared to the control (Table 3). 

**Fig. 4:**
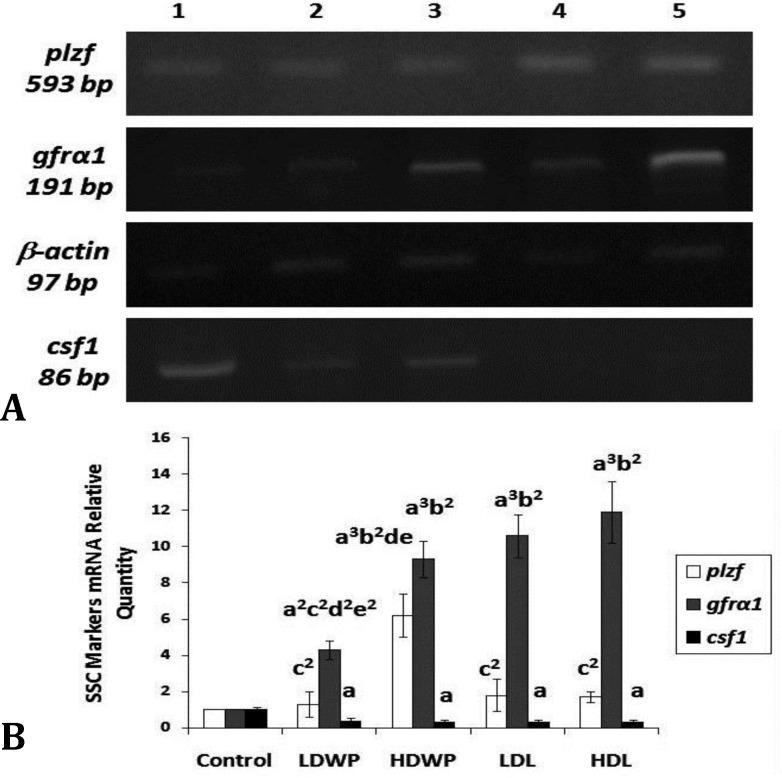
RT-PCR analysis of mRNA level of spermatogonial stem cells markers in control and treated groups.** A)** Representative agarose gel electrophoresis of RT-PCR products of control and treated rats. Lane 1: Control; lane 2: LDWP; lane 3: HDWP; lane 4: LDL; lane 5: HDL. **B)** Relative quantity of PLZF, GFRα1, and CSF1 mRNA in treated groups in comparison to the control group, values were normalized to β-actin. Values are expressed as mean ± SD, n=10 in each experiment, three independent experiments.  a: *p*   0.05; a^2^: *p*   0.01; a^3^: *p*   0.001 versus control, b^2^: *p*   0.01 versus LDWP, c^2^: *p*   0.01 versus HDWP, d: *p*   0.05; d^2^: *p*   0.01 versus LDL, e: *p*   0.05 ; e^2^: *p* < 0.01 versus HDL

**The aqueous extract of** ***T. officinale*** **whole plant or leaves have counteracting effects on the expression of GRFα1 and CFS1 spermatogonial stem cell markers** **.** The PLZF mRNA level was significantly increased only in the HDWP-treated group when compared with the control and treated groups (Figs. 4A and 4B). The mRNA level of GFRΑ1 was increased in all of the treated groups when compared with the control, and in the HDWP- and the leaves-treated groups when compared with the LDWP- receiving groups (Figs. 4A and 4B). On the other hand, CSF1 mRNA level was decreased in all of the treated groups as compared with the control (Figs. 4A and 4B). 

**Table 3:** Effect of *T. officinale* treatment on testicular histology of control and treated groups. Values are expressed as mean ± SD, n = 10 in each group

**Groups**	**Seminiferous tubule diameter ** **(µm)**	**Germinal epithelium thickness ** **(µm)**	**Interstitial space** ** (µm)**	**Number of Leydig cells ** **(per 200 ** **µm** ^2^ **)**
**Control**	2510.60 ± 11.90	70.40 ± 3.10	31.10 ± 2.20	8.10 ± 1.60
**LDWP**	188.10 ± 25.10a3^c^^d^e2	48.30 ± 4.90a2^c^^d^e2	41.90 ± 4.10a2	6.20 ± 1.80^a^
**HDWP**	127.50 ± 12.10a3^b^	28.20 ± 2.80a3^b^	46.20 ± 4.80a3	5.20 ± 1.20^a^
**LDR**	159.60 ± 13.20a3^b^	34.90 ± 9.910a3^b^	54.20 ± 3.90a3	4.50 ± 1.60a2
**HDR**	136.50 ± 17.90a3b3	26.00 ± 1.300a3b2	58.30 ± 5.30a3	3.70 ± 1.50a2

LDWP: Low dose-receiving whole plant; HDWP: High dose-receiving whole plant; LDL: Low dose-receiving leaves; HDL: High dose-receiving leaves.

a : *p* 0.05;

a2 : *p* 0.01;

a3 : *p* 0.001 compared to control,

b *p* 0.05;

b2 *p* 0.01;

b3 : *p* 0.001 compared to LDWP,

c : *p* 0.05 compared to HDWP,

d : *p* 0.05 compared to LDL,

e2 : *p* < 0.01 compared to HDL.

**Fig. 5:**
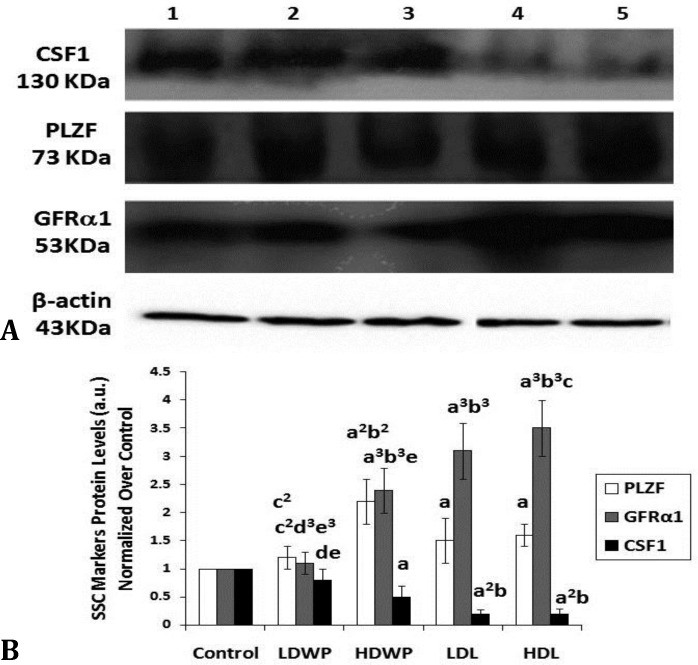
*Taraxacum officinale* treatment leads to changes in protein levels of spermatogonial stem cells.** A) **Representative Western blot of testicular lysates of control and treated rats blotted for spermatogonial stem cell markers CSF1; PLZF; and GFR 1. β-actin was used as a loading marker. Lane 1: Control; lane 2: LDWP; lane 3: HDWP; lane 4: LDL; lane 5: HDL. The experiment was repeated three times and the corresponding quantification is shown in (B). **B)** Quantification of CSF1, PLZF, and GFRα1 protein levels (normalized to actin) in control and treated rats. a: *p*   0.05; a^2^: *p*   0.01; a^3^: *p*   0.001 compared to control, b: *p*   0.05; b^2^: *p*   0.01; b^3^: *p*   0.001 compared to LDWP, c: *p*   0.05; c^2^: *p*   0.01 compared to HDWP, d: *p*   0.05; d^3^: *p*   0.001 compared to LDL, e: *p*   0.05 ; e^3^: *p* < 0.001 compared to HDL

The PLZF protein level was increased in the HDWP- and the leaves-treated groups when compared with the control group, and in the HDWP-treated groups when compared with the LDWP-receiving group (Figs. 5A and 5B). The CSF1 protein level was decreased in all of the treated groups, except the LDWP-receiving group, when compared with the control group and in the leaves-receiving groups as compared to the LDWP-receiving group (Figs. 5A and 5B). The protein level of GFR1 was increased in all treated groups, except the LDWP-receiving group, when compared with the control and the LDWP- receiving groups (Figs. 5A and 5B).

## Discussion

*Taraxacum officinale* has been well-known for its medicinal properties in different cultures. It has been described to treat illnesses such as anemia, cirrhosis of the liver, hepatitis, inflammation, and cancer. It is used in Jordan folk medicine for the treatment of male infertility.^22^ However, a recent study has proved that *T. officinale* whole plant aqueous extract decreases male fertility.^12^ Thus, the present study was conducted to investigate if *T. officinale* leaves aqueous extract has the similar anti-spermatogenic activities and whether these activities are mediated through spermatogonial stem cells. 

Treating male rats with the aqueous extract of *T. officinale* did not cause a change in body weight (Table 1). This result is in agreement with Takzare *et al*. who observed a similar effect of the aqueous extract of *Achillea millefolium* (Compositae) when administered to rats.^23^ In addition, the weight of the kidney and liver of *T. officinale-*treated rats were not significantly different when compared with the control group (Table 1). Similar effects were reported for *Echinacea purpurea* (Compositae),^24^ and *T. officinale *leaves aqueous extract.^25^

The results of the present study revealed that the oral administration of *T. officinale *whole plant or leaves aqueous extract causes a decrease in testes weight, except in LDWP-receiving group, when compared with the control (Table 1). These results are compatible with the results of Yakubu who showed a decrease in testis weight of male rats after the treatment with *Chromolaena odorata* (Compositae).^26^ The author attributed this reduction in testis weight to a decrease in the testosterone level. 

Spermatogenesis is a complex process regulated by different hormones such as testosterone, FSH and LH. Testosterone is secreted by Leydig cells and it exerts its effects by binding to the androgen receptor (AR) located in Sertoli and myoid cells. Testosterone is crucial in the maintenance of spermatogenesis.^27^ The treatment of rats with *T. officinale* extract for 60 days caused reduction in the number of Leydig cells/unit area of the interstitial tissues (Fig. 3 and Table 3); this in turn caused a decrease in testosterone level in all treated groups (Table 2). 

Improved sperm quality (motility, morphology and count) plays a major role in the enhancement of pregnancy rate and the number of fetuses delivered.^28^ In this study, the impaired sperm functions in *T*. *officinale*-treated groups (Table 2) caused a reduction in pregnancy rate and in the number of fetuses delivered in all of the treated groups, except for LDWP-receiving group. This result is in accordance with Agarwal *et al*. study which showed that the oral administration of *Calendula officinalis* (Compositae) to male rats caused a decrease in pregnancy rate.^29^

Spermatogenesis is initiated at puberty when spermato-gonia differentiate to sperms in the seminiferous tubules of the testis.^30^ In this study, seminiferous tubules sections prepared from testes of control rats showed highly organized germinal epithelium structure and normal spermatogenesis (Figs. 3A and 3B). However, the oral administration of *T. officinale *whole plant aqueous extract caused arrest of spermatogenesis at spermatid stage (late maturation arrest) and disorganization of germ cells (Figs. 3C and 3D). In addition, it caused hypoplasia of germinal epithelium and Leydig cells (Table 3). The testicular sections prepared from the leaves-receiving rats exhibited similar testicular histology to the whole plant-receiving rats (Figs. 3E and 3F). This result is in agreement with Padashetty and Mishra’s study which revealed that the extract of *Echinops echinatus* (Compositae) caused dis-organization in the architecture of the seminiferous tubular germinal epithelium, and decreased the seminiferous tubular diameter.^31^ The reason for this was explained as a significant decrease in testosterone level similar to what we have found in the current study (Tables 2 and 3).

The GDNF/GFRα1 signaling pathway plays a major role in the maintenance of undifferentiated SSCs, and their over-expression leads to accumulation of clusters of undifferentiated spermatogonia and testicular tumors.^6^^,^^32^ In the current study, the expression level of GFRα1 was increased in the treated groups when compared with the control group (Figs. 4 and 5). In addition, and to a lesser extent, the expression level of PLZF was increased in the treated groups, except for the LDWP-receiving group, as compared with the control (Figs. 4 and 5). The PLZF expression is restricted to the undifferentiated spermato-gonia, and it is crucial for the regulation of SSCs self-renewal; PLZF represses spermatogonial differentiation.^6^^,^^33^ Costoya *et al*. showed that the decrease in PLZF expression lead to progressive loss of spermatogonia with age.^34^ The expression level of CSF1 was decreased in all of the treated groups; this was not surprising since the sources of CSF1, the cells of the interstitial tissue, were hypoplastic as evident by the reduction of the number of Leydig cells/unit area of the interstitial connective tissues (Fig. 3 and Table 3). 

Taken together, the over-expression of GFRα1 in all of the treated groups accompanied with the down regulation CSF1 could explain the histology of the seminiferous tubules and the results of the fertility parameters. Spermatogonial stem cells self-renewal and differentiation signals must be balanced at a 1:1 ratio.^32^ *Taraxacum officinale* whole plant or leaves aqueous extract treatment tipped the balance towards the self- renewal side but not to the degree where only spermatogonia can be seen; rather the effects were exhibited as late maturation arrest, lower sperm count, lower pregnancy rate and lower number of fetuses. 

In conclusion, the oral administration of the aqueous extract of the whole plant or leaves of *T. officinale *have similar anti-fertility effects. Both extracts decreased sperm count, motility, and pregnancy rate, and both caused structural changes in testicular tissue such as loss of germ cells, late spermatogenesis arrest and reduction in the number of Leydig cells. The changes in the gene expression of the SSCs markers (PLZF, CSF1 and GFRα1) led to the imbalance between spermatogonia self-renewal and differentiation leading consequently to the late maturation arrest noticed in the seminiferous tubules of treated groups. Based on these results, further studies are essential to determine which step(s) in spermiogenesis is affected by *T. officinale* treatment thus causing late maturation arrest.
